# Research prospects for kidney xenotransplantation: a bibliometric analysis

**DOI:** 10.1080/0886022X.2023.2301681

**Published:** 2024-02-23

**Authors:** Shujun Yang, Mingtao Zhang, Hao Wei, Bin Zhang, Jiang Peng, Panfeng Shang, Shengkun Sun

**Affiliations:** a Department of Urology, Lanzhou University Second Hospital, Lanzhou, China; b Department of Orthopaedics, Lanzhou University Second Hospital, Lanzhou, China; c Department of Urology, Qingdao University Hospital, Qingdao, China; d Department of Orthopaedics, Chinese PLA General Hospital, Chinese PLA Medical School, Beijing, China; e Department of Urology, Chinese PLA General Hospital, Chinese PLA Medical School, Beijing, China

**Keywords:** Xenotransplantation, xenograft, renal, kidney, bibliometric

## Abstract

**Background:**

Xenograft kidney transplantation has been receiving increasing attention. The purpose of this study is to use bibliometric analysis to identify papers in this research field and explore their current status and development trends.

**Methods:**

Using the data in the Web of Science core database from Clarivate Analytics as the object of study, we used ‘TS = Kidney OR Renal AND xenotransplantation’ as the search term to find all literature from 1980 to 2 November 2022.

**Results:**

In total, 1005 articles were included. The United States has the highest number of publications and has made significant contributions in this field. Harvard University was at the forefront of this study. Professor Cooper has published 114 articles in this field. Xenotransplantation has the largest number of relevant articles. Transplantation was the most cited journal. High-frequency keywords illustrated the current state of development and future trends in xenotransplantation. The use of transgenic pigs and the development of coordinated co-stimulatory blockers have greatly facilitated progress in xenotransplantation research. We found that ‘co-stimulation blockade’, ‘xenograft survival’, ‘pluripotent stem cell’, ‘translational research’, and ‘genetic engineering’ were likely to be the focus of attention in the coming years.

**Conclusions:**

This study screened global publications related to xenogeneic kidney transplantation; analyzed their literature metrology characteristics; identified the most cited articles in the research field; understood the current situation, hot spots, and trends of global research; and provided future development directions for researchers and practitioners.

## 1. Introduction

Chronic kidney disease (CKD) currently affects 850 million people worldwide, and is expected to become one of the most important causes of death beyond the mid-twenty-first century as people live longer [1]. In Europe, the population with end-stage renal disease (ESRD) is estimated to be over one million and varies greatly from country to country [2]. In the United States (US), the number of patients waiting for a kidney transplant exceeds one million; however, only a very small number of patients receive transplant treatment [3–5]. Advances in kidney transplantation have improved ESRD treatment. A shortage of organs due to insufficient kidney sources is the biggest obstacle in transplantation [6]. The first attempt at kidney xenotransplantation was made by Princeteau et al. in 1905, who placed thinly sliced rabbit kidney tissue under the peritoneum of a uremic patient [7]. Since the start of kidney xenotransplantation, the survival time of genetically modified pig kidneys in monkeys has been extended, reaching 758 days, which has opened up a new direction for organ transplantation [8–10].

The main obstacle in xenotransplantation is the reactivity of natural human antibodies to several carbohydrate xenoantigens expressed in pig cells. In recent years, clustered randomly interspaced short palindromic repeats and associated protein 9 (CRISPR/Cas9) gene editing technology has solved this problem [11–13], which includes the absence of known carbohydrate heterologous antigen expression and the expression of human gene fragments [14]. In addition, kidney grafts can survive for a long time with the use of continuously optimized immunosuppressive, anti-inflammatory, and adjunctive drug regimens used in pig to non-human primates (NHPs) kidney xenotransplantation, making it possible to transform this study into a clinical trial [15,16]. Montgomery et al. [17] transplanted kidneys of transgenic pigs into two brain-dead patients. In the 54-h study, the transplanted kidney remained pink, well perfused, and continued to produce urine throughout the study, and the biopsy did not show hyperacute rejection (HR).

Using bibliometric analysis, we can obtain the research status, hotspots, and future research directions in this field [18]. At present, bibliometric analyses of many oncology and immunology studies have emerged in an endless stream [19,20], which have played a guiding role in the research in corresponding fields and have enabled scholars to gain a deeper understanding of the development of each field. A literature search revealed that although there are many studies in the field of renal xenotransplantation, there is no corresponding bibliometric analysis of the trends, countries, authors, keywords, or citations in this field. What are the current research hotspots and future research trends of kidney xenotransplantation? Have rejection reactions been resolved, can long-term survival be achieved, how can controversial ethical issues be resolved, and can it move toward clinical practice? These issues are not known. Therefore, we used the corresponding analysis software to conduct the first bibliometric analysis of kidney xenotransplantation to summarize the research status and hot topics in this field.

## 2. Methods

### 2.1. Data sources and searches

**Figure 1.**
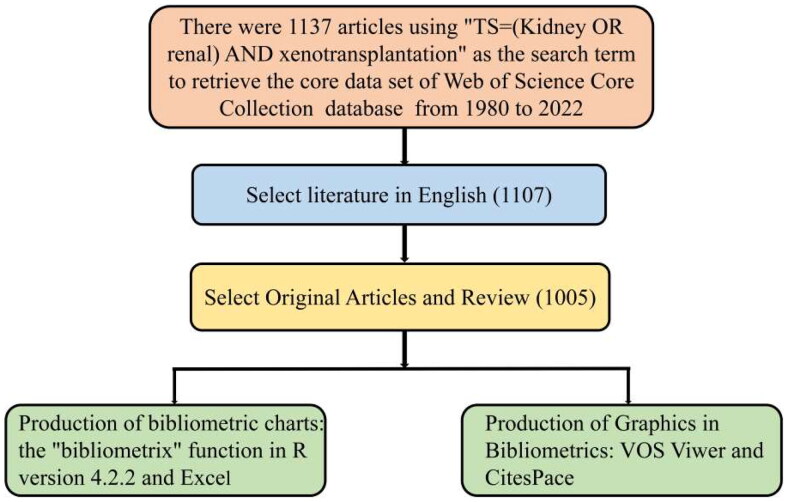
Flowchart.

We used the data in the Web of Science core database from Clarivate Analytics as the object of study. This high-quality digital literature resource database has been accepted by a wide range of researchers and is considered the most suitable database for bibliometric analyses. We used ‘TS = (kidney OR renal) AND xenotransplantation’ as the search term to find all literature from 1980 to 2 November 2022. Selecting article and review by written in English, the flowchart is shown in Figure 1. This study was conducted in accordance with the guidelines of the Declaration of Helsinki.

### 2.2. Data analysis and visualization

Two independent researchers conducted the study to ensure the reliability of the results. The retrieved documents were downloaded in ‘plain text’ format and the corresponding information was extracted for data analysis. Author, country or region, institution, keyword, and reference data were analyzed and plotted using both VOSviewer 1.6.17 and CiteSpace 5.8. R3: Literature analysis software. The ‘bibliometrics’ function in R4.2.2 (R Foundation for Statistical Computing, Vienna, Austria, http://www.R-project.org/) was used to import the data and analyze the collaboration between countries and the research hotspots in the neighborhood. Microsoft Excel (Redmond, WA) was used to analyze the volume of articles issued by country or region and graphically show trends in the number of articles issued.

## 3. Results

### 3.1. Annual publications and trends

**Figure 2.**
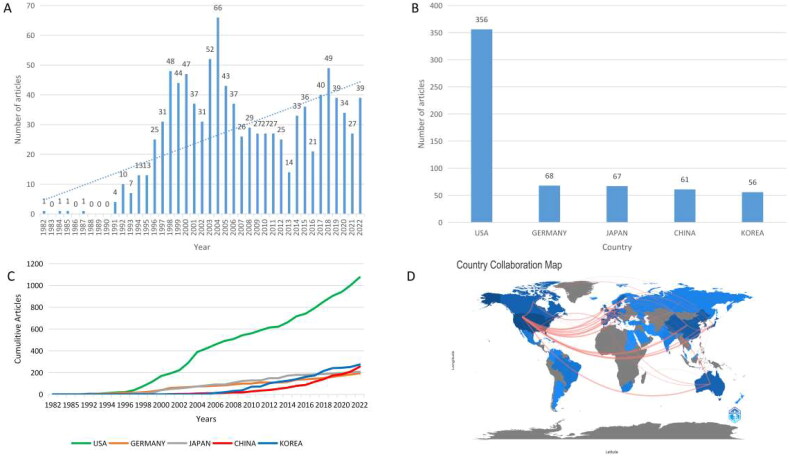
Trends and countries of issuance.

The studies included were 834 articles (83.28%) and 171 reviews (17.01%). The literature covers 47 countries/regions and 656 institutions. Research on kidney xenotransplantation from 1982 to 2022 has increased, with an increasing number of publications. The annual number of publications from 1982 to 2022 is shown in Figure 2(A) and was divided into four phases: slow growth period (1982–1990), first peak period (1991–2012), second peak period (2013–2020), and rapid growth period (2021–2022). Before 1990, the number of publications grew relatively slowly, which then grew rapidly after 1991, with more than four publications per year, and reached a peak in 2004, with 66 articles in one year. The number of publications gradually increased to a peak and then decreased during 2013–2020; and in the last 2 years, the number of publications showed an increasing trend.

### 3.2. Issuance by country/regions and institutions

**Figure 3.**
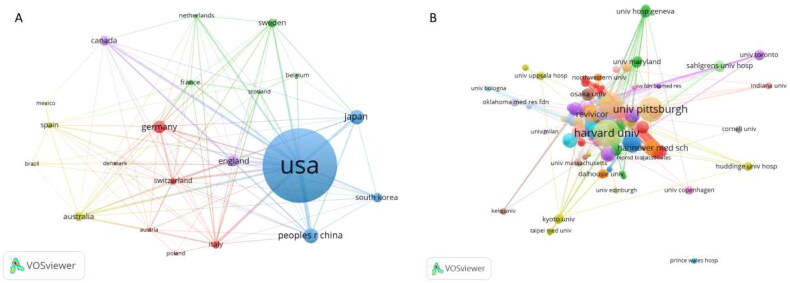
Visual analysis of the sending country and organization.

A total of 47 countries or regions published this type of research, and the top five countries or regions in terms of the number of articles published are shown in Figure 2(B). The US had the highest number of publications, followed by Germany, Japan, China, and South Korea. China has seen a dramatic increase in the number of articles published in recent years and was expected to overtake Korea as the second-most published country after this year (Figure 2(C)). Figure 1(D) clearly shows that the US has the most frequent partnerships with various countries, a phenomenon that is highlighted in European countries, China, Australia, and others. The partnerships of all countries are shown in Figures 3(A) and 2(D). The size of the circle indicates the number of texts published, and the thickness of the line indicates the closeness of the connection between two parties. Among the 656 issuing institutions, the top 10 institutions belong to five countries, but six are from the US: Harvard University, The University of Alabama at Birmingham, The University of Pittsburgh, Washington University, Columbia University, and the Mayo Clinic. We also found that the institutions with the highest number of publications did not match the top five countries in terms of the number of publications, as we know that Japan and China have more publications, but the institutions are scattered. The linkages among all the institutions are shown in Figure 3(B). The largest issuing institution is Harvard University, which is closely associated with all the institutions. The numbers and proportions of the top 10 issuing countries and institutions are listed in Table 1.

**Table 1.** The top 10 countries and institutions in the kidney xenotransplantation.

Rank	Country	Count (%)	Institution	Count (%)
1	USA	356 (35.4%)	Harvard University	124 (12.3%)
2	Germany	68 (6.8%)	Seoul National University	90 (9.0%)
3	Japan	67 (6.7%)	The University of Alabama at Birmingham	84 (8.4%)
4	China	61 (6.1%)	University of Pittsburgh	73 (7.3%)
5	Korea	56 (5.6%)	Washington University	70 (7.0%)
6	United Kingdom	48 (4.8%)	Columbia University	47 (4.7%)
7	Sweden	47 (4.7%)	University of Padua	47 (4.7%)
8	Canada	41 (4.1%)	Hannover Medical School	44 (4.4%)
9	Australia	39 (3.9%)	The University of Western Ontario	38 (3.8%)
10	Spain	37 (3.7%)	Mayo clinic	33 (3.3%)

### 3.3. Analysis of journals and co-cited journals

**Figure 4.**
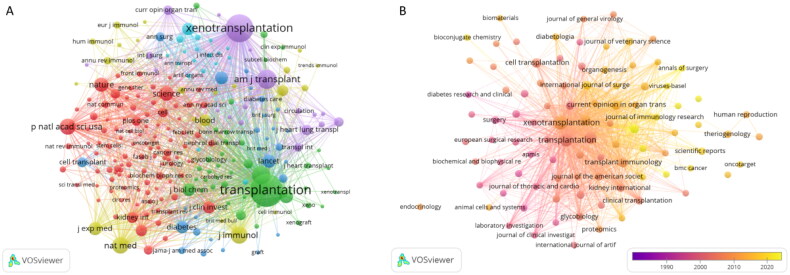
Visual analysis of journals and co-cited journals.

Articles published as of November 2022 were published in 310 journals. The largest proportion of articles was published in *Xenotransplantation* (*n* = 232, 23.1%), followed by *Transplantation* (*n* = 132, 13.1), *Transplantation Proceedings* (*n* = 66, 6.6%), *American Journal of Transplantation* (*n* = 36, 3.6%), and *Current Opinion in Organ Transplantation* (*n* = 19, 1.9%) (Table 2). Among the top 15 journals with the highest number of kidney allograft publications, the top three journals with the highest impact factors (IFs) were *Kidney International* (IF = 19.00), *International Journal of Surgery* (IF = 13.40), and the *American Journal of Transplantation* (IF = 9.37) (Figure 4(A)). Of the 15 journals, 73% were classified as Q2 and above, and one was Q4 (Table 2). Of the top 15 co-cited journals on kidney allografts, *Transplantation* (6147 citations) was the most frequently cited, followed by *Xenotransplantation* (3923 citations), and the *American Journal of Transplantation* (1531 citations). Moreover, of these 15 journals, all were Q2 and above, except for *Transplantation Proceedings* and *Progress in Transplantation*, which were Q4 (Table 3, Figure 4(B)).

**Table 2.** The top 15 journals in the kidney xenotransplantation (2021).

Rank	Journal	Count	IF	*Q* (JCR)
1	*Xenotransplantation*	232 (23.1%)	3.79	2
2	*Transplantation*	132 (13.1%)	5.39	1
3	*Transplantation Proceedings*	66 (6.6%)	1.01	4
4	*American Journal of Transplantation*	36 (3.6%)	9.37	1
5	*Current Opinion in Organ Transplantation*	19 (1.9%)	2.27	3
6	*Cell Transplantation*	17 (1.7%)	4.14	2
7	*Transplant Immunology*	17 (1.7%)	2.03	3
8	*Transplant International*	11 (1.1%)	3.84	2
9	*Frontiers in Immunology*	10 (1.0%)	8.79	1
10	*International Journal of Surgery*	9 (0.9%)	13.4	1
11	*Clinical Transplantation*	8 (0.8%)	3.46	3
12	*Kidney International*	8 (0.8%)	19.0	1
13	*PLOS One*	8 (0.8%)	3.24	2
14	*Glycobiology*	7 (0.7%)	5.96	2
15	*Journal of Immunology*	7 (0.7%)	5.43	2

**Table 3.** Top 15 co-cited journals for research of the kidney xenotransplantation (2021).

Rank	Journal	Co-citation	IF	*Q* (JCR)
1	*Transplantation*	6147	5.39	1
2	*Xenotransplantation*	3923	3.79	2
3	*American Journal of Transplantation*	1531	9.37	1
4	*Transplantation Proceedings*	1301	1.01	4
5	*Journal of Immunology*	1094	5.43	2
6	*Nature Medicine*	1034	87.24	1
7	*Proceedings of the National Academy of Sciences of the United States of America*	911	12.78	1
8	*Science*	744	63.71	1
9	*Nature*	679	69.50	1
10	*Progress in Transplantation*	629	1.07	4
11	*Journal of Experimental Medicine*	584	17.58	1
12	*Lancet*	543	202.73	1
13	*Journal of Biological Chemistry*	517	5.47	2
14	*New England Journal of Medicine*	496	176.08	1
15	*Journal of Clinical Investigation*	443	19.46	1

### 3.4. Analysis of authors and co-cited authors

**Figure 5.**
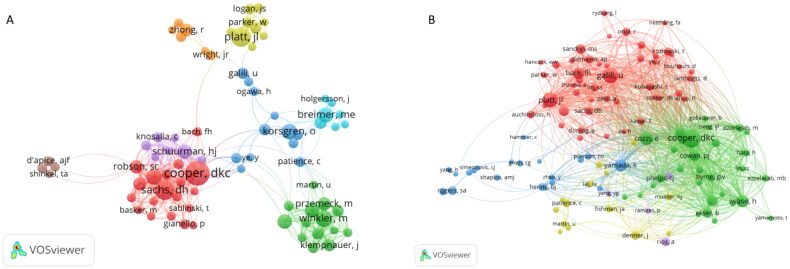
Visual analysis of authors and co-cited authors.

From all authors who have published literature related to renal allografts in the years 1982–2022, the 10 most influential authors are listed in Table 4. The author who published the most kidney transplantation papers was Cooper (*n* = 114), followed by Sanchs (*n* = 44) and Hara (*n* = 38). The network of authors contributing to and co-citing kidney xenotransplantation is shown in Figure 5(A). The most prominent nodes were associated with the authors who posted the most articles and co-cited authors. The three most co-cited authors were Cooper, Sanchs, and Awwad (Figure 5(B)). In addition, we found that Cooper and Sanchs ranked first and second in both authorship and co-cited authorship, indicating that these two individuals were the most active within the field.

**Table 4.** Ranking of the top 10 authors and co-cited authors in the kidney xenotransplantation.

Rank	Authors	Count	Co-cited authors	Citations
1	Cooper DCK	114	Cooper DCK	1531
2	Sachs DH	44	Sachs DH	853
3	Hara H	38	Awwad M	570
4	Ayares D	35	Robson SC	485
5	Iwase H	30	Ayares D	399
6	Platt JL	29	Hara H	384
7	Cozzi E	28	Buhler L	352
8	Yamada K	27	Cozzi E	339
9	Robson SC	23	Yamada K	339
10	Schuurman HJ	23	Shimizu A	309

### 3.5. Analysis co-cited references

**Figure 6.**
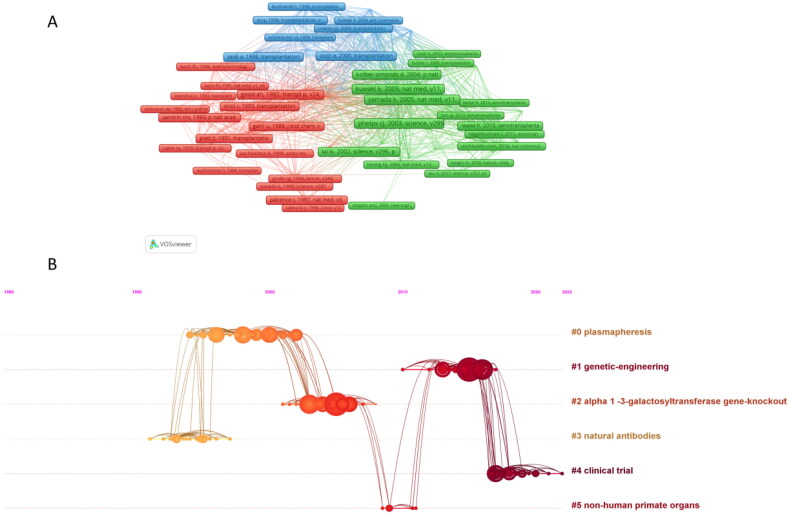
Visual analysis of co-cited references.

A visualization of the co-cited literature was constructed using the size of the nodes and the strength of the connecting lines (Figure 6(A)). The 10 most frequently cited articles are shown in Table 5, where the most frequently cited article on ‘α-1,3-galactosy transferase (GT) knockout mini-pigs (GTKO pigs)’ by Phelps et al. can be seen (with 111 co-citations) [21].

**Table 5.** The top 10 co-cited references related to colorectal cancer immunotherapy [21–30] (2021).

Rank	Title	Citations	Journal IF	Quartile in category
1	Production of alpha 1,3-galactosyltransferase-deficient pigs	111	*Science* (IF = 63.80)	Q1
2	Marked prolongation of porcine renal xenograft survival in baboons through the use of alpha1,3-galactosyltransferase gene-knockout donors and the cotransplantation of vascularized thymic tissue	110	*Nature Medicine* (IF = 87.24)	Q1
3	Heart transplantation in baboons using alpha1,3-galactosyltransferase gene-knockout pigs as donors: initial experience	102	*Nature Medicine* (IF = 87.24)	Q1
4	Identification of carbohydrate structures that bind human antiporcine antibodies: implications for discordant xenografting in humans	93	*Transplantation Proceedings* (IF = 1.01)	Q4
5	The generation of transgenic pigs as potential organ donors for humans	91	*Nature Medicine* (IF = 87.24)	Q1
6	Production of alpha-1,3-galactosyltransferase knockout pigs by nuclear transfer cloning	81	*Science* (IF = 63.80)	Q1
7	Production of alpha-1,3-galactosyltransferase null pigs by means of nuclear transfer with fibroblasts bearing loss of heterozygosity mutations	79	*Proceedings of The National Academy of Sciences of The United States of America* (IF = 12.78)	Q1
8	Immunopathology of hyperacute xenograft rejection in a swine-to-primate model	70	*Transplantation* (5.39)	Q2
9	Man, apes, and old world monkeys differ from other mammals in the expression of alpha-galactosyl epitopes on nucleated cells	69	*The Journal of Biological Chemistry* (5.49)	Q2
10	Anti-pig IgM antibodies in human serum react predominantly with Gal(alpha 1–3)Gal epitopes	65	*Proceedings of The National Academy of Sciences of The United States of America*(IF = 12.78)	Q2

Additionally, we performed a co-citation analysis of the literature on a timeline (Figure 6(B)). We found that ‘natural antibodies’ (cluster 3) was the earliest hotspot; ‘plasmapheresis’ (cluster 0), ‘GT’ (cluster 2), ‘genetic engineering’ (cluster 3), and ‘knockout’ (cluster 2) were mid-term (1995–2005) research hotspots; and ‘genetic engineering’ (cluster 1) and ‘clinical trial’ (cluster 5) have been the most studied in recent years (2010–2022) and are the current new hotspots in this field of renal allografts.

**Figure 7.**
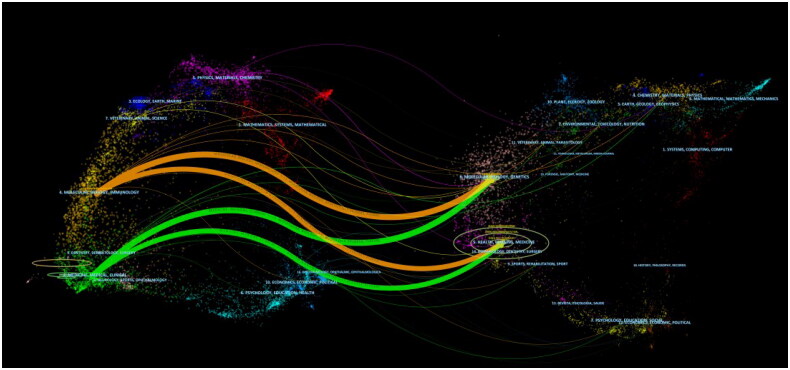
Double graph superposition of periodicals.

Double-map superposition of journals describes the distribution and direction of topics in academic journals. This curve is the cited curve, which indicates the source of the subject. The research on ‘molecular/biology/immunology’ and ‘medicine/medical/clinical’ comes from ‘molecular/biology/genetics and health/nursing/medicine’ (Figure 7).

### 3.6. Analysis of keywords and burst keywords

**Figure 8.**
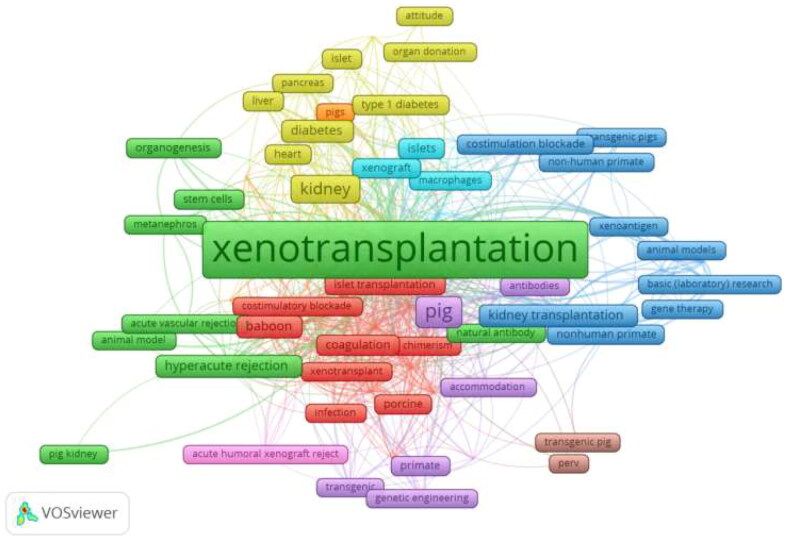
Visual analysis of keyword.

We analyzed the frequency and link strength of the keywords using the VOS viewer software. When the minimum number of occurrences of a keyword was five, there were 102 keywords. We then set the threshold value to 6, and there were 77 keyword occurrences. The keyword with the highest frequency was xenotransplantation (429), followed by pigs (92), kidneys (58), transplantation (36), and immunosuppression (29) (Figure 8).

**Figure 9.**
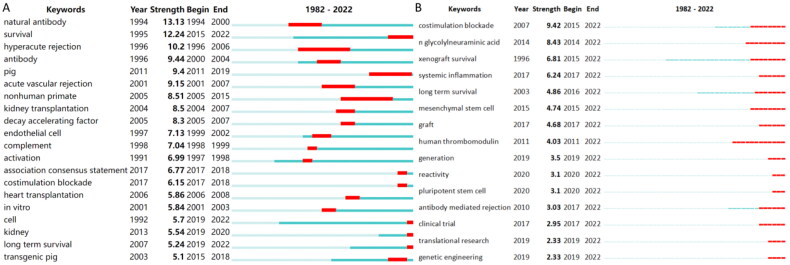
Analysis of keyword burst.

To identify research frontiers in the field, CiteSpace was used to find burst keywords. In the top 129 keywords with the strongest citation bursts, we focused on keywords that have burst in recent years (Figure 9), including ‘co-stimulation blockade’ (burst intensity of 9.42), ‘n glycolylneuraminic acid’ (burst intensity of 8.43), ‘xenograft survival’ (burst intensity of 6.81), ‘systemic inflammation’ (burst intensity of 6.24), ‘long term survival’ (burst intensity of 4.86), ‘mesenchymal stem cell’ (burst intensity of 4.74), ‘graft’ (burst intensity of 4.68), ‘human thrombomodulin’ (burst intensity of 4.03), ‘generation’ (burst intensity of 3.5), ‘reactivity’ (burst intensity of 3.1), ‘pluripotent stem cell’ (burst intensity of 3.1), ‘antibody-mediated rejection’ (burst intensity of 3.03), ‘clinical trial’ (burst intensity of 2.95), ‘translational research’ (burst intensity of 2.33), and ‘genetic engineering’ (burst intensity of 2.33).

## 4. Discussion

### 4.1. Exploration of basic information

We obtained 1005 papers related to kidney xenotransplantation published between 1982 and 2022 for analysis using the Scientific Core Database. There was a general trend toward a gradual increase in the number of publications. By analyzing the number of national and regional publications, we found that the US is the clear leader [31], not only with 35% of publications, but also in all aspects of the number of collaborations with other countries and the frequency of citations. In addition, China has gradually increased its number of publications since 2006 and is likely to overtake Korea as the second-most published country by 2023 [32].

Of the 10 institutions with the highest number of publications worldwide, 60% belong to the US, indicating that the US has played an important and leading role in the academic development of the field. Harvard University holds the top spot with the highest number of publications and articles cited, and it also has the highest average number of citations (53.9). This phenomenon indicates the high quality of Harvard publications and the pivotal role they play in the field. Although China and Japan rank in the top five in terms of the number of documents issued worldwide, their sending agencies have not entered the top 10 in the world. Thus, the sending agencies of China and Japan are relatively decentralized, and there is less interagency cooperation.

Cooper (*n* = 114) published the most papers on kidney xenotransplantation, followed by Sanchs (*n* = 44); these two authors are consistent among the co-cited authors, indicating that they are the most influential in the field of kidney xenotransplantation. As of November 2022, 1005 articles have appeared in 310 journals. Most documents were sent *via Xenotransplantation* (*n* = 232, 23.1%) followed by *Transplantation* (*n* = 132, 13.1%). Among the top 15 journals with the largest number of papers, *Kidney International* (IF = 19.00) and the *International Journal of Surgery* (IF = 13.40) had an IF of more than 10 points (Table 1). Among the first 15 co-cited journals on kidney xenotransplantation, *Transplantation* (6147) was cited the most, followed by *Xenotransplantation* (3923). Therefore, researchers can better understand the progress in this field and identify potential collaborators by reading the articles published in these journals.

According to the timeline chart, cited documents can be classified into six categories that can be roughly divided into three stages: early, middle, and current. It can be seen from the analysis that the early research mainly focused on ‘plasmapheresis’ and ‘natural antibodies’; the mid-term research mainly focuses on ‘GT’ and ‘non-human primitive organs’; and the current research focuses on ‘genetic engineering’ and ‘clinical trial’. By analyzing the top 10 most cited articles, we can find that the word ‘GT’ appears repeatedly in the title. This study has been the most important in the field of xenotransplantation in the past decades [33].

By using bibliometrics to analyze keyword bursts, we can understand the hot topics and scope of academic discussion in this research field. We used the top 20 keywords with the strongest citation bursts to analyze hot and cutting-edge topics and divided the interval into three periods (early, middle, and current). Early research topics included natural antibodies, HR, antibodies, acute vascular rejection, endothelial cells, complement activation, and *in vitro* studies. With the evolution of time and the development of medical technology, the hot topics of mid-term research became NHP, kidney xenotransplantation, decay-accelerating factors, and heart transplantation. The current hot topics of research are survival, pigs, association consensus statements, costimulation blockade, cells, kidneys, long-term survival, and transgenic pigs. The use of transgenic pigs in kidney xenotransplantation has become a major research trend [14]. Overcoming ethical problems and rejection, and ensuring long-term survival in the pig–non-primate transplantation model remain hot topics in current research [34]. Among the top 129 keywords that have been cited the most frequently in recent years, ‘generation’, ‘reactivity’, ‘pluripotent stem cell’, ‘translational research’, and ‘genetic engineering’ appeared after 2019 and continue to the present. These theme words are current and may become future hot topics.

### 4.2. Frontiers of porcine-primate transplantation models

Genome editing has greatly promoted the progress of kidney xenotransplantation. The results of this study showed that the use of GTKO pigs in xenotransplantation is a major finding [35–37]. In 2002, the journal *Science* reported that Lai et al. successfully bred GTKO pigs by nuclear transplantation of heterozygous mutant-deficient fibroblasts. This approach cleared GT gene expression and offered hope for overcoming HR of xenografts. Gene editing has become increasingly sophisticated and has enabled the editing of multiple genes [38]. Commonly used editing protocols include gene deletion, knockout of three porcine carbohydrate antigens and porcine growth hormone gene receptor genes, and targeted insertion of human complement inhibitor genes (hDAF and hCD46), human anticoagulation genes (hTBM and hEPCR), and immunomodulatory genes (hCD47 and hHO1) [39–44].

### 4.3. Costimulation blockade

The use of costimulation blockers has replaced previous medication regimens and is a new breakthrough. Yamamoto demonstrated that blockade of the CD40:CD154 costimulatory pathway was superior to immunosuppressive therapy and that the outcome remained better in kidneys transplanted with six gene-edited species [45]. A tacrolimus-based immunotherapy regimen was administered to baboons that received conventional treatment. Graft failure is primarily caused by an antibody-mediated rejection [34]. In contrast, for baboons receiving anti-CD154 antibody-based [46] or anti-CD40 protocols, the experiments were terminated mainly because of infection or other complications [47].

### 4.4. Long term survival and clinical research

Long-term survival after xenotransplantation remains a focus of current and future research. In 1963, researchers transplanted a kidney from a chimpanzee into a dying 23-year-old male with renal failure, and the recipient was administered immunosuppressive treatments, such as azathioprine, prednisone, and total body irradiation. Ultimately, this patient survived for 9 months, which is the longest surviving xenotransplantation experiment in humans [17]. Thus, xenotransplantation will soon be translated into clinical trials. This is a major research direction of our study [48,49].

### 4.5. Ethical issues

Ethical issues in xenotransplantation have received widespread attention from different perspectives. Gene-edited pigs as a source of kidneys in kidney transplants can reduce the illegal trade in organs, and have the advantage of eliminating the risks associated with the removal of kidneys from healthy altruistic living donors [50,51]. However, there are also many controversies [52–55]: Will the genetically modified kidney function in the human environment persist for a long time after transplantation, even if it disrupts the normal physiological functions of the body? What are the psychological and physical effects on recipients? If the patient produces offspring after transplantation, is the next generation is affected [56]. This is a major research direction of our study. Ultimately, kidney allografts are beneficial for patients with multiple diseases that necessitate allogeneic transplantation. These issues have been the subject of a long-standing debate regarding renal allograft transplantation. Ultimately, kidney xenotransplantation provides additional benefits for patients with multiple diseases. It is hoped that kidney transplants will be implemented soon to help more patients [7, 57].

### 4.6. Possible future research trends and models

Summarizing and analyzing the above data, the future research trends and models in the field of xenograft kidney transplantation are listed as follows: (1) the success of preclinical research on kidney xenotransplantation is a necessary condition for conducting clinical trials. Therefore, gene edited pig to NHP kidney xenotransplantation models are still the main and long-term research content in various institutions [9]; (2) which gene editing scheme is most suitable for kidney xenotransplantation is still in the exploratory stage; (3) the application of co stimulatory pathway blockers in future clinical trials of kidney xenotransplantation needs to be validated for their effectiveness; (4) in recent years, there has been a significant increase in attention on whether transplanted kidneys can function normally, such as maintaining the renin angiotensin aldosterone system and electrolyte balance, which are important issues [51, 53, 58,59]; (5) issues such as biosafety, virus infection, and monitoring are also worth exploring and solving; (6) the conduct of clinical trials will provide more reliable data for the clinical application of xenograft kidney transplantation.

### 4.7. Advantages and disadvantages

Due to its small size and limited quantity, keyword indexes are not comprehensive and have limitations; the analyzed data were only retrieved from WOS and included English article research, which means our analysis may be incomplete. But the results of this bibliometric analysis are reliable and reproducible.

## 5. Conclusions

Kidney xenotransplantation could be the ultimate solution to the worldwide shortage of kidney donor organs; therefore, breaking through research in this area would be the next great revolutionary advance. With the maturation of multiple knockouts and gene transfers in transgenic pigs, and the use of novel immunosuppressive agents in a porcine-non-primate transplantation model, the survival time of this model has continued to break through and be guaranteed. Clinical kidney xenotransplantation trials will become a reality in the near future.

## References

[1] Jager KJ, Kovesdy C, Langham R, et al. A single number for advocacy and communication-worldwide more than 850 million individuals have kidney diseases. Nephrol Dial Transplant. 2019;34(11):1–11. doi: 10.1093/ndt/gfz174. 31566230

[2] Kramer A, Pippias M, Noordzij M, et al. The European Renal Association – European Dialysis and Transplant Association (ERA-EDTA) Registry Annual Report 2015: a summary. Clin Kidney J. 2018;11(1):108–122. doi: 10.1093/ckj/sfx149. 29423210 PMC5798130

[3] Porrett PM, Orandi BJ, Kumar V, et al. First clinical-grade porcine kidney xenotransplant using a human decedent model. Am J Transplant. 2022;22(4):1037–1053. doi: 10.1111/ajt.16930. 35049121

[4] Carrier AN, Verma A, Mohiuddin M, et al. Xenotransplantation: a new era. Front Immunol. 2022;13:900594. doi: 10.3389/fimmu.2022.900594. 35757701 PMC9218200

[5] Wijkstrom M, Iwase H, Paris W, et al. Renal xenotransplantation: experimental progress and clinical prospects. Kidney Int. 2017;91(4):790–796. doi: 10.1016/j.kint.2016.08.035. 27914702 PMC5357451

[6] Yu X-H, Deng W-Y, Jiang H-T, et al. Kidney xenotransplantation: recent progress in preclinical research. Clin Chim Acta. 2021;514:15–23. doi: 10.1016/j.cca.2020.11.028. 33301767

[7] Rodger D, Cooper D. Kidney xenotransplantation: future clinical reality or science fiction? Nurs Health Sci. 2022;25(1):161–170. doi: 10.1111/nhs.12994. 36335558 PMC10124775

[8] Adams AB, Lovasik BP, Faber DA, et al. Anti-C5 antibody tesidolumab reduces early antibody-mediated rejection and prolongs survival in renal xenotransplantation. Ann Surg. 2021;274(3):473–480. doi: 10.1097/SLA.0000000000004996. 34238812 PMC8915445

[9] Anand RP, Layer JV, Heja D, et al. Design and testing of a humanized porcine donor for xenotransplantation. Nature. 2023;622(7982):393–401. 37821590 10.1038/s41586-023-06594-4 PMC10567564

[10] Firl DJ, Lassiter G, Hirose T, et al. Clinical and molecular correlation defines activity of physiological pathways in life-sustaining kidney xenotransplantation. Nat Commun. 2023;14(1):3022. doi: 10.1038/s41467-023-38465-x. 37311769 PMC10264453

[11] Söllner J-H, Sake HJ, Frenzel A, et al. In vitro genome editing activity of Cas9 in somatic cells after random and transposon-based genomic Cas9 integration. PLOS One. 2022;17(12):e0279123. doi: 10.1371/journal.pone.0279123. 36584049 PMC9803249

[12] Cowan PJ, Hawthorne WJ, Nottle MB. Xenogeneic transplantation and tolerance in the era of CRISPR–Cas9. Curr Opin Organ Transplant. 2019;24(1):5–11. doi: 10.1097/MOT.0000000000000589. 30480643

[13] Watanabe S, et al. The combinational use of CRISPR/Cas9 and targeted toxin technology enables efficient isolation of bi-allelic knockout non-human mammalian clones. Int J Mol Sci. 2018;19(4):1075. 29617297 10.3390/ijms19041075 PMC5979347

[14] Niu D, Ma X, Yuan T, et al. Porcine genome engineering for xenotransplantation. Adv Drug Deliv Rev. 2021;168:229–245. doi: 10.1016/j.addr.2020.04.001. 32275950

[15] Fishman JA, Sachs DH, Yamada K, et al. Absence of interaction between porcine endogenous retrovirus and porcine cytomegalovirus in pig-to-baboon renal xenotransplantation in vivo. Xenotransplantation. 2018;25(5):e12395. doi: 10.1111/xen.12395. 29624743 PMC6158079

[16] Cowan PJ, Robson SC. Progress towards overcoming coagulopathy and hemostatic dysfunction associated with xenotransplantation. Int J Surg. 2015;23(Pt B):296–300. doi: 10.1016/j.ijsu.2015.07.682. 26220018

[17] Montgomery RA, Stern JM, Lonze BE, et al. Results of two cases of pig-to-human kidney xenotransplantation. N Engl J Med. 2022;386(20):1889–1898. doi: 10.1056/NEJMoa2120238. 35584156

[18] Jiang D, Ji T, Liu W, et al. Four decades of clinical liver transplantation research: results of a comprehensive bibliometric analysis. Transplantation. 2022;106(10):1897–1908. doi: 10.1097/TP.0000000000004224. 35831925

[19] Baghban N, Ullah M, Nabipour I. The current trend of exosome in epithelial ovarian cancer studies: a bibliometric review. Front Pharmacol. 2023;14:1082066. doi: 10.3389/fphar.2023.1082066. 36969852 PMC10034012

[20] Guo K, Li J, Li X, et al. Emerging trends and focus on the link between gut microbiota and type 1 diabetes: a bibliometric and visualization analysis. Front Microbiol. 2023;14:1137595. doi: 10.3389/fmicb.2023.1137595. 36970681 PMC10033956

[21] Phelps CJ, Koike C, Vaught TD, et al. Production of alpha 1,3-galactosyltransferase-deficient pigs. Science. 2003;299(5605):411–414. 12493821 10.1126/science.1078942 PMC3154759

[22] Yamada K, Yazawa K, Shimizu A, et al. Marked prolongation of porcine renal xenograft survival in baboons through the use of alpha1,3-galactosyltransferase gene-knockout donors and the cotransplantation of vascularized thymic tissue. Nat Med. 2005;11(1):32–34. doi: 10.1038/nm1172. 15619627

[23] Kuwaki K, Tseng Y-L, Dor FJMF, et al. Heart transplantation in baboons using alpha1,3-galactosyltransferase gene-knockout pigs as donors: initial experience. Nat Med. 2005;11(1):29–31. doi: 10.1038/nm1171. 15619628

[24] Good AH, et al. Identification of carbohydrate structures that bind human antiporcine antibodies: implications for discordant xenografting in humans. Transplant Proc. 1992;24(2):559–562. 1566430

[25] Cozzi E, White DJ. The generation of transgenic pigs as potential organ donors for humans. Nat Med. 1995;1(9):964–966. doi: 10.1038/nm0995-964. 7585226

[26] Lai L, Kolber-Simonds D, Park K-W, et al. Production of alpha-1,3-galactosyltransferase knockout pigs by nuclear transfer cloning. Science. 2002;295(5557):1089–1092. doi: 10.1126/science.1068228. 11778012

[27] Kolber-Simonds D, Lai L, Watt SR, et al. Production of alpha-1,3-galactosyltransferase null pigs by means of nuclear transfer with fibroblasts bearing loss of heterozygosity mutations. Proc Natl Acad Sci U S A. 2004;101(19):7335–7340. doi: 10.1073/pnas.0307819101. 15123792 PMC409919

[28] Platt JL, Fischel RJ, Matas AJ, et al. Immunopathology of hyperacute xenograft rejection in a swine-to-primate model. Transplantation. 1991;52(2):214–220. doi: 10.1097/00007890-199108000-00006. 1871792

[29] Galili U, Shohet SB, Kobrin E, et al. Man, apes, and old world monkeys differ from other mammals in the expression of alpha-galactosyl epitopes on nucleated cells. J Biol Chem. 1988;263(33):17755–17762. doi: 10.1016/S0021-9258(19)77900-9. 2460463

[30] Sandrin MS, Vaughan HA, Dabkowski PL, et al. Anti-pig IgM antibodies in human serum react predominantly with gal(alpha 1–3)gal epitopes. Proc Natl Acad Sci U S A. 1993;90(23):11391–11395. doi: 10.1073/pnas.90.23.11391. 7504304 PMC47988

[31] Jagdale A, Cooper DKC, Iwase H, et al. Chronic dialysis in patients with end-stage renal disease: relevance to kidney xenotransplantation. Xenotransplantation. 2019;26(2):e12471. doi: 10.1111/xen.12471. 30456901 PMC6476633

[32] Wang Y, Lei T, Wei L, et al. Xenotransplantation in China: present status. Xenotransplantation. 2019;26(1):e12490. doi: 10.1111/xen.12490. 30740782

[33] Cooper D, Pierson RR. Milestones on the path to clinical pig organ xenotransplantation. Am J Transplant. 2023;23(3):326–335. doi: 10.1016/j.ajt.2022.12.023. 36775767 PMC10127379

[34] Sykes M, Sachs DH. Progress in xenotransplantation: overcoming immune barriers. Nat Rev Nephrol. 2022;18(12):745–761. doi: 10.1038/s41581-022-00624-6. 36198911 PMC9671854

[35] Lee W, Hara H, Ezzelarab MB, et al. Initial in vitro studies on tissues and cells from GTKO/CD46/NeuGcKO pigs. Xenotransplantation. 2016;23(2):137–150. doi: 10.1111/xen.12229. 26988899 PMC4842123

[36] Firl DJ, Markmann JF. Measuring success in pig to non-human-primate renal xenotransplantation: systematic review and comparative outcomes analysis of 1051 life-sustaining NHP renal allo- and xeno-transplants. Am J Transplant. 2022;22(6):1527–1536. doi: 10.1111/ajt.16994. 35143091

[37] Yamada K, Sykes M, Sachs DH. Tolerance in xenotransplantation. Curr Opin Organ Transplant. 2017;22(6):522–528. doi: 10.1097/MOT.0000000000000466. 28937406 PMC5737964

[38] Mohiuddin MM, Singh AK, Corcoran PC, et al. Chimeric 2C10R4 anti-CD40 antibody therapy is critical for long-term survival of GTKO.hCD46.hTBM pig-to-primate cardiac xenograft. Nat Commun. 2016;7(1):11138. doi: 10.1038/ncomms11138. 27045379 PMC4822024

[39] Abicht J-M, Sfriso R, Reichart B, et al. Multiple genetically modified GTKO/hCD46/HLA-E/hbeta2-mg porcine hearts are protected from complement activation and natural killer cell infiltration during ex vivo perfusion with human blood. Xenotransplantation. 2018;25(5):e12390. doi: 10.1111/xen.12390. 29536572

[40] Hara H, Long C, Lin YJ, et al. In vitro investigation of pig cells for resistance to human antibody-mediated rejection. Transpl Int. 2008;21(12):1163–1174. doi: 10.1111/j.1432-2277.2008.00736.x. 18764834

[41] Jagdale A, Nguyen H, Li J, et al. Does expression of a human complement-regulatory protein on xenograft cells protect them from systemic complement activation? Int J Surg. 2020;83:184–188. doi: 10.1016/j.ijsu.2020.09.034. 32987208 PMC7686296

[42] Ekser B, Rigotti P, Gridelli B, et al. Xenotransplantation of solid organs in the pig-to-primate model. Transpl Immunol. 2009;21(2):87–92. doi: 10.1016/j.trim.2008.10.005. 18955143

[43] Lee KFE, Lu B, Roussel JC, et al. Protective effects of transgenic human endothelial protein C receptor expression in murine models of transplantation. Am J Transplant. 2012;12(9):2363–2372. doi: 10.1111/j.1600-6143.2012.04122.x. 22681753

[44] Takeuchi K, Ariyoshi Y, Shimizu A, et al. Expression of human CD47 in pig glomeruli prevents proteinuria and prolongs graft survival following pig-to-baboon xenotransplantation. Xenotransplantation. 2021;28(6):e12708. doi: 10.1111/xen.12708. 34418164 PMC8957703

[45] Yamamoto T, Hara H, Foote J, et al. Life-supporting kidney xenotransplantation from genetically engineered pigs in baboons: a comparison of two immunosuppressive regimens. Transplantation. 2019;103(10):2090–2104. 10.1097/TP.0000000000002796 31283686

[46] Cooper D. Recent progress in the pig-to-nonhuman primate kidney transplantation model: report of a symposium. Xenotransplantation. 2022;29(1):e12728. doi: 10.1111/xen.12728. 35001421

[47] Iwase H, Hara H, Ezzelarab M, et al. Immunological and physiological observations in baboons with life-supporting genetically engineered pig kidney grafts. Xenotransplantation. 2017;24(2). doi: 10.1111/xen.12293. PMC5397334 28303661

[48] Reese PP, Parent B. Promoting safety, transparency, and quality in xenotransplantation. Ann Intern Med. 2022;175(7):1032–1034. doi: 10.7326/M22-0539. 35576589

[49] Fischer K, Schnieke A. Xenotransplantation becoming reality. Transgenic Res. 2022;31(3):391–398. doi: 10.1007/s11248-022-00306-w. 35545691 PMC9135885

[50] Cowan PJ, Cooper DK, D’Apice AJ. Kidney xenotransplantation. Kidney Int. 2014;85(2):265–275. doi: 10.1038/ki.2013.381. 24088952 PMC3946635

[51] Iwase H, Klein EC, Cooper DK. Physiologic aspects of pig kidney transplantation in nonhuman primates. Comp Med. 2018;68(5):332–340. doi: 10.30802/AALAS-CM-17-000117. 30208986 PMC6200029

[52] DeLaura I, Anwar IJ, Ladowski J, et al. Attitudes of patients with renal disease on xenotransplantation: a systematic review. Xenotransplantation. 2023;30(2):e12794. doi: 10.1111/xen.12794. 36880602

[53] Hansen-Estruch C, Cooper D, Judd E. Physiological aspects of pig kidney xenotransplantation and implications for management following transplant. Xenotransplantation. 2022;29(3):e12743. doi: 10.1111/xen.12743. 35297098 PMC9232961

[54] Rodger D, Hurst DJ, Cooper DK. Xenotransplantation: a historical-ethical account of viewpoints. Xenotransplantation. 2023;30(2):e12797. doi: 10.1111/xen.12797. 36943143 PMC10101926

[55] Hawthorne WJ, Thomas A, Pierson RN. Ethics and theoretical issues in kidney xenotransplantation. Semin Nephrol. 2022;42(4):151288. doi: 10.1016/j.semnephrol.2022.151288. 36587995

[56] Cengiz N, Wareham CS. Ethical considerations in xenotransplantation: a review. Curr Opin Organ Transplant. 2020;25(5):483–488. doi: 10.1097/MOT.0000000000000796. 32833703

[57] Tatapudi VS, Griesemer AD. Physiologic considerations of pig-to-human kidney xenotransplantation. Curr Opin Nephrol Hypertens. 2023;32(2):193–198. doi: 10.1097/MNH.0000000000000858. 36683545

[58] Hansen-Estruch C, Bikhet MH, Javed M, et al. Renin–angiotensin–aldosterone system function in the pig-to-baboon kidney xenotransplantation model. Am J Transplant. 2023;23(3):353–365. doi: 10.1016/j.ajt.2022.11.022. 36695679 PMC10124771

[59] Hansen-Estruch C, Bikhet MH, Shaik IH, et al. Assessment of glomerular filtration and tubular secretion in baboons with life-supporting pig kidney grafts. Xenotransplantation. 2023;30(2):e12795. doi: 10.1111/xen.12795. 36820525 PMC10354795

